# Pharmacists’ Approach to Optimise Safe Medication Use in Paediatric Patients

**DOI:** 10.3390/pharmacy9040180

**Published:** 2021-11-03

**Authors:** Nicole Keuler, Annatjie Bouwer, Renier Coetzee

**Affiliations:** 1 School of Pharmacy, University of the Western Cape, Cape Town 7535, South Africa; recoetzee@uwc.ac.za; 2 Centralized Monitoring Solutions, IQVIA, Bloemfontein 9301, South Africa; annatjie.bouwer@iqvia.com

**Keywords:** paediatric patients, paediatric medicine, pharmacist, medication errors

## Abstract

Paediatric patients are unique, yet challenging patients to care for by pharmacists. Paediatric medicine use requires special consideration. Pharmacists play an important role in educating and counselling patients, carers, and healthcare workers. Further, pharmacists have the necessary knowledge and skills to optimise safe medicine use in paediatric patients. This article provides basic principles for safe practices in paediatric medicine by following the nine rights of medication administration.

## 1. Introduction

Incorrect dosing of medicines is a frequently reported medication error [1,2,3,4]. A South African study conducted in a public sector tertiary hospital in 2017 reported that 78% of patients in the paediatric and neonatal intensive care units were exposed to at least one medication error [5]. In the US, approximately 30% of medication errors occur in the paediatric population [1]. Close to 40% of parents or guardians (referred to as ‘carers’ throughout) make an error when administering liquid formulations to their child [6].

Medication errors are associated with healthcare professionals’ knowledge and training, patient characteristics, environmental factors, prescribing errors, and lack of communication [7]. To optimise medication safety in paediatric patients, a multidisciplinary, integrated approach is critical. Pharmacists play a significant role in optimising medication safety in paediatric patients and ensuring better patient outcomes [8,9].

Here, we focus on pharmacists’ role and their ability to optimise their approach to paediatric medicine and patient care in hospital and community settings.

A ‘medication error’ is a preventable event that occurs during any step of the medication use process (i.e., prescribing, preparing, dispensing, and administering) [10,11,12]. However, when these errors are not prevented, it can lead to potentially harmful adverse events. A paediatric patient in the intensive care unit is at a higher risk for medication errors since they are prescribed numerous medicines to treat severe diseases. These conditions require intravenous therapy, which often needs dilution and/or reconstitution [13]. Medicines that are associated with errors in paediatric patients include anti-infectives, electrolytes and fluids, analgesics, sedatives, and proton pump inhibitors [2,14].

## 2. Paediatric Dosing Challenges

Paediatric patients are a unique patient group that pharmacists serve. They require greater attention, vigilance, and special care due to the complexity of paediatric dosing [1,8]. Medication administration by carers could be challenging and might be influenced by various factors. Lack of counselling, inappropriate measuring equipment, and literacy levels have been associated with administration errors [6].

Further, paediatric patients require age-appropriate formulations (i.e., formulations that deliver an accurate dose), which are safe and acceptable to children while reducing medication errors. Paediatric-friendly formulations are limited, making safe medicine use challenging. The lack of paediatric-specific formulations often forces healthcare providers to use medicine off-label, resulting in medication errors [11,12]. Preference is given to liquid formulations due to easy administration compared to tablets and capsules. Tablets and capsules present various challenges when administering the prescribed dose to paediatric patients since tablets need to be either crushed or dissolved, and capsules need to be opened and dissolved [11].

## 3. Nine Rights Principles

Five rights of medication prescription have been identified to reduce medication errors [15]; however, this could be interpreted further. The right principles of prescribing and administering medication could be adapted to the nine rights of medication administration [15,16]:

The right medicine and right formulation for the right patient for the right indication at the right dose using the right measuring equipment to administer the medicine through the right route at the right time for the right duration.

When reviewing the prescription, the pharmacist should review the nine rights of prescribing medication. The nine rights principles will be discussed in a systematic approach that can be followed when prescribing or reviewing a prescription for a paediatric patient.

### 3.1. Right Medicine and Right Formulation

Before a medicine is prescribed, the formulation available should be considered to ensure safe and convenient dosing. A pharmacist working in the ward or present during a ward round discussion might advise on the most appropriate medicinal product and formulation.

Liquid formulations are preferred in paediatric patients as these are easier to administer and reduce the risk of medication administration errors [17,18]. Controlled and extended-release medicines (e.g., sodium valproate CR) as well as enteric-coated medicines (omeprazole) should not be crushed due to their special formulation; therefore, they require special consideration. Crushing these tablets would compromise medication stability and might lead to treatment failure. Enteric-coated formulations are formulated to be protected in the stomach due to instability in acid environments. Crushing the tablets might lead to gastric irritation, destruction of content by stomach acid, and premature onset of action. Extended-release products are formulated to release the content over a prolonged period, and crushing the tablets might lead to immediate release, which would increase the risk of toxicity [18]. Depending on the prescribed medicine, the formulation should be changed to an immediate-release medicine formulation (e.g., carbamazepine tablets instead of controlled-release tablets), another formulation (e.g., carbamazepine or sodium valproate syrup instead of controlled-release tablets) [18], or another medicine that might be more appropriate and better tolerated. If the recommendations are not possible, appropriate administration techniques should be shared with the carer. This is particularly important with the administration of lansoprazole capsules (open and dissolved content of capsule in apple juice) and omeprazole tablets (dissolved in apple juice). With these extemporaneous products, it is important to counsel the carer to make a fresh solution for each dose prescribed due to the instability of the prepared product [18].

When patients cannot swallow, medicines may need to be administered through a nasogastric (NG) tube. However, medication stability might be compromised when administered in this manner (e.g., phenytoin adsorbs the NG tube) [18]. If medicines need to be administered through an NG tube, alternatives routes (e.g., intravenous or intramuscular) should be considered to optimise medication therapy. Liquid formulations are preferred but dispersible tablets might also be used depending on product availability and patient-specific characteristics [18,19]. Alternative routes reduce the risk of treatment failure. The NG tube should be flushed with 15 to 30 mL of water before and after administration to prevent tube blockage and to ensure that all the medicines are administered to optimise medicine availability [19].

When preparing medicines, it is important to be vigilant of look- or sound-alike products as this can lead to medication errors [20]. Examples of these products are:

- Adco-Magnesium^®^, Adco-Metoclopramide^®^, and Adco-Furosemide^®^;
- Sandoz Co-Amoxyclav SF^®^ 250 mg/5 mL and Sandoz Co-Amoxyclav S^®^ 125 mg/5 mL;
- Ampicillin Fresenius^®^ 500 mg and Ampicillin Fresenius^®^ 250 mg;
- Adco-Ipratropium^®^ and Adco-Fenoterol^®^.

### 3.2. Right Patient

Always confirm the patient’s identity and patient-specific prescription chart. Confirm at least two patient identifiers with the carer/healthcare worker that worked with the patient previously (e.g., patient name and address) [21]. Other patient characteristics to consider include age, weight, allergies, ability to swallow, and fluid restriction.

#### 3.2.1. Age and Weight

The paediatric patient’s age is important to ensure age-appropriate medicine use. Therefore, the patient’s paediatric term must be known (i.e., neonate, term, preterm neonate, infant, or child) when prescribing and calculating the appropriate dose. Table 1 summarises the paediatric-related terms. The paediatric patient’s age also guides the choice of the product formulation and could easily be calculated by reviewing the date of birth. Drops and liquid formulations are preferred in preterm and term neonates, infants and toddlers, and even pre-school children. Tablets or chewable tablets are preferred in pre-school and school children [3]. Dose recommendations for paediatric patients are based on milligram per kilogram; therefore, weight is critical when calculating the appropriate dose [22].

#### 3.2.2. Allergies

Note all allergies on the prescription to ensure safe medicine use. Good history-taking is paramount since a previous allergic response guides the prescriber in future prescriptions. Further, differentiate between a true allergy and sensitivity reaction [26].

#### 3.2.3. Ability to Swallow

Certain paediatric patients (e.g., cerebral palsy patients) have difficulty swallowing; thus, liquid formulations or drops are easier to administer, might reduce medicine administration errors [3], and increase patient compliance.

#### 3.2.4. Fluid Restriction

Often paediatric patients are fluid-restricted, e.g., preterm infants. Then, it is recommended to prepare the prescribed medicines using the least possible volume of diluent (e.g., trimethoprim 400 mg/sulfamethoxazole 80 mg per 5 mL in 75 mL of 5% dextrose) [27] or using the highest concentration product (e.g., amoxicillin 250 mg/5 mL). Daily medication review is critical in these patients.

### 3.3. Right Indication

The indication for the prescribed medicine is crucial, especially with antibiotics, and should be noted on the prescription chart [28]. Dose recommendations differ based on the indication, e.g., meropenem is given at higher doses (40 mg/1 kg) in the case of meningitis and at lower doses, 10 and 20 mg/1 kg, for complicated skin and skin structure infections or intra-abdominal infections, respectively [29]. Another example is cefalexin; the recommended daily dosage is 25 to 50 mg/1 kg per day in divided doses, while in severe infections, the dosage should be increased to 75 to 100 mg/1 kg per day in four divided doses [30]. The dose of cotrimoxazole for the prevention of opportunistic infections and the treatment of *Pneumocystis* pneumonia also differs [27]. Medication interactions should be considered with polypharmacy in the paediatric population. Daily review of prescriptions should be part of ward round discussions to reduce adverse effects and medicine interactions and to ensure rational medicine use [12].

### 3.4. Right Dose

Calculating the correct dose is crucial to ensure safe and effective medicine use and to prevent under- (treatment failure) and/or overdosing (toxicity) of medicines in paediatric patients. Kidney and hepatic function as well as weight are patient-specific factors to consider when calculating the correct dose. Additionally, therapeutic drug monitoring (TDM) optimises medication doses while preventing adverse effects. To ensure that the correct dose is administered with intravenous medicines, the concepts of reconstitution and dilution need to be understood.

#### 3.4.1. Reconstitution versus Dilution

Intravenous medicines could be given as a bolus or an infusion. Certain medicines require dilution while others are administered undiluted [29]. These preparation methods should be individualised and re-considered with available generic or new products. Certain medicines require reconstitution with a diluent to be administered as a bolus but can further be diluted to be administered as an infusion [29]. Understanding the difference ensures that the correct dose is administered.

#### 3.4.2. Calculating the Dose

Appropriate use of measuring equipment and convenient dosing should also be considered [17,31] to:

- assist in easy administration for carers/healthcare workers;
- reduce administration errors; and
- prevent over- and underdosing of paediatric patients.

Liquid formulations might be easier to administer and should be used if available instead of tablets in children under the age of five years [17,32].

For example, an infant might weigh 4.2 kg, and the recommended dosage is 30 mg/1 kg/dose every 8 h. Thus, the equation would be:

(1) $$\mathrm{dosage}\;\mathrm{to}\;\mathrm{be}\;\mathrm{administered}\;\left(x\right)=4.2\;\mathrm{kg}\;\times \;30\;\mathrm{mg}=126\;\mathrm{mg}\;\mathrm{per}\;\mathrm{dose}$$

However, amoxicillin is available as a 125 mg/5 mL product; therefore, 5 mL per 125 mg is easier to administer.

The prescriber should be vigilant when prescribing the dosage, as some references recommend dosages based on milligram per kilogram per dose to be administered multiple times a day or milligram per kilogram per day in divided doses. In a challenging environment, mistakes can occur. To prevent medication errors, pharmacists could review paediatric patients’ doses through a daily prescription review. Dose calculations for prescribing or administering should be critically evaluated and calculated with vigilance. If there is any uncertainty about the dose, the reference should be consulted to prevent prescription or administration errors [21]. 

When calculating the dose, the active component of the medicine needs to be considered, especially in combination products (e.g., piperacillin/tazobactam and amoxicillin/clavulanic acid) as well as the different strengths of the products available (e.g., 125 and 250 mg/5 mL or milligram per millilitre or milligram per 5 mL). Additionally, it is important to ensure that the dose prescribed and the strength of the product are in the same units (e.g., mg/µg/g).

The equations below can be used to calculate the dose of prescribed medicines [21].

(2) $$\mathrm{dosage}\;\mathrm{to}\;\mathrm{be}\;\mathrm{administered}\;\left(x\right)=\frac{\mathrm{dosage}\;\mathrm{prescribed}\;\mathrm{in}\;\mathrm{mg}}{\mathrm{strength}\;\mathrm{of}\;\mathrm{product}\;\mathrm{available}\;\mathrm{in}\;\mathrm{mg}}\;\times \;\frac{\mathrm{strength}\;\mathrm{of}\;\mathrm{product}\;\mathrm{available}\;\mathrm{in}\;\mathrm{mL}}{1}$$

or

(3) $$\mathrm{dosage}\;\mathrm{to}\;\mathrm{be}\;\mathrm{administered}\;\left(x\right)=\left[\frac{\mathrm{dosage}\;\mathrm{prescribed}\;(\mathrm{mg})}{\mathrm{strength}\;\mathrm{of}\;\mathrm{product}\;(\mathrm{mg})}\right]\times \;\mathrm{strength}\;\mathrm{of}\;\mathrm{product}\;(\mathrm{mL})$$

Table 2 shows common medicines prescribed in infants with calculation pearls.

Examples of dosage equations based on different strengths of the available product in milligram are:

(4) Rx amoxicillin/clavulanic acid (Augmentin^®^) = 375 mg per 8 h
Available product: Augmentin
125 mg (amoxicillin)/31.25 mg (clavulanic acid)/5 mL suspension = (375 mg/125 mg) × 5 mL = 15 mL

(5) Rx amoxicillin/clavulanic acid (Augmentin^®^) = 375 mg per 8 h
Available product: Augmentin
250 mg (amoxicillin)/62.5 mg (clavulanic acid)/5 mL suspension = (375 mg/250 mg) × 5 mL = 7.5 mL

Note the strength in milligram per millilitre of the product, e.g., paracetamol is available in 120 mg/5 mL and ferrous gluconate in 8 mg/1 mL.

Equation (6) provides an example of dosage calculation based on milligram per millilitre of product available.

(6) Rx paracetamol = 360 mg per 6 h
Available product: paracetamol (120 mg/5 mL) = (360 mg/120 mg) × 5 mL = 15 mL
Rx ferrous gluconate = 60 mg per od
Available product: ferrous gluconate (8 mg/1 mL) = (60 mg/8 mg) × 1 mL = 7.5 mL

Equation (7) provides an example of dosage calculation based on the active ingredient in a combination product.

(7) Rx amoxicillin/clavulanic acid (Augmentin^®^) = 375 mg per 8 h
Available product: Augmentin
125 mg (amoxicillin)/31.25 mg (clavulanic acid)/5 mL suspension = (375 mg/125 mg) × 5 mL = 15 mL
Dosage based on amoxicillin component.
Rx cotrimoxazole = 20 mg per 6 h
Available product: sulfamethoxazole (200 mg)/trimethoprim (40 mg)/5 mL = (20 mg/40 mg) × 5 mL = 2.5 mL
Dosage based on trimethoprim component.

Often tablets need to be crushed and dissolved. Considerations for the dosage calculations include tablet strength if the tablet is scored and the volume of diluent to administer a convenient dose (see Equation (8)).

(8) Rx spironolactone = 3.125 mg per 12 h
Available product: spironolactone (25 mg) (can be halved since it is scored)
Using half, a tablet dissolved in 5 mL = (3.125 mg/12.5 mg) × 5 mL = 1.25 mL
Using half, a tablet dissolved in 10 mL = (3.125 mg/12.5 mg) × 10 mL = 2.5 mL

#### 3.4.3. Kidney Function

Medicines that are renally cleared might require dose adjustments (e.g., amikacin) or should be avoided when the kidneys’ function is impaired (e.g., ibuprofen). This concept should be considered when prescribing anti-tuberculosis (TB) or antiretroviral therapy (ART) in renal impairment [18,35]. Table 3 summarises medicines that are cleared by the kidneys.

#### 3.4.4. Hepatic Function

Hepatically cleared medicines might require dose adjustments if a patient has liver impairment, while other medicines should be avoided, such as paracetamol for paracetamol toxicity. This should also be considered when prescribing anti-TB or ART in liver impairment [18,35]. Table 4 lists medicines that require dose adjustment in patients with hepatic impairment.

#### 3.4.5. TDM

Antibiotics such as vancomycin and aminoglycosides require TDM to guide the prescriber to ensure safe and effective medicine use. Other medicines that require TDM to prevent toxicity and to ensure treatment efficacy include anti-epileptic medicines (carbamazepine, phenytoin, and sodium valproate) [18].

### 3.5. Right Measuring Equipment

To prevent medication errors, appropriate measuring equipment should be used. Measuring cups have increasingly caused medication errors [1,11] compared to syringes, which are more user-friendly. Various medicinal equipment (e.g., medicine spoons and syringes) are available for administering oral medicines. The prescribed dose should be considered when choosing the appropriate measuring equipment. A medicine spoon (5 mL) might be used for children; however, in neonates or infants, a syringe might be more appropriate to administer the dose with ease and to reduce medication errors [17,31].

### 3.6. Right Route of Administration

Medicines could be administered orally, intravenously, intramuscularly, rectally, subcutaneously, through nebulising, or through an NG tube. The route of administration should be legible on the prescription and confirmed before administration. The route of administration should also be clearly communicated with the carer.

### 3.7. Right Time

Medicine could be administered every day, 12 h, 8 h, 6 h, or more frequently, depending on the product’s indication and half-life. Medicines should be administered at the prescribed time to ensure safe and effective medicine use. Further, it is important that carers understand the meaning of the dosing intervals, e.g., “three times a day” means the medicine should be administered every 8 h. Additional information about when the medicine should be taken should be shared with the carer (e.g., should be taken on an empty stomach or after food). For example, omeprazole should be administered before food, while amoxicillin/clavulanic acid should preferably be taken after food to alleviate gastrointestinal side effects.

### 3.8. Right Duration

Each medicine prescribed should have an indicated duration. This is important when prescribing antimicrobials. Pharmacists are stewards of antimicrobials in the hospital and community; they optimise antimicrobial use in paediatric patients and ensure rational antibiotic use to reduce resistance [36,37,38]. In hospital settings, prescription charts should be reviewed daily to ensure that the medicine is still indicated. In community settings, monthly reviews of prescribed medicines should be considered. Again, it is vital that carers understand the duration of medicine use.

## 4. Medication Storage

Storing medicine correctly plays a role in the safety of medicines for paediatric patients. Certain products require refrigeration after preparation, while others are stable at room temperature after preparation. The package insert and label should always be consulted to confirm storage requirements. Expiry dates of medicines should be checked before administering to ensure safe and effective medicine use [21]. This practice could prevent the use of slow-moving items that might expire on the shelf.

## 5. Pharmacists’ Role in Paediatric Medicine

Pharmacists could improve paediatric patient care, irrespective of the level of care, e.g., hospital or community setting [11]. Pharmacists are equipped with the necessary skills (calculation of doses/dosages, detail-orientated, communication skills, and identifying medication-related errors) and knowledge (pharmacology and pharmaceutics) to improve paediatric medicine use.

Pharmacists could prevent medication errors through the practice of certain daily activities [1,8]. Medication reconciliation is a unique service that pharmacists could incorporate in their daily activities when reviewing a paediatric patient’s prescription to reduce medication discrepancies [14]. During this process, previous medicines prescribed should be compared with the current prescription to identify medication-related problems (MRPs) and to optimise medication therapy. Over-the-counter medicine use, e.g., a salbutamol inhaler, should be part of the medication review process as overuse might indicate uncontrolled asthma. The practice of medication reconciliation should be applied at the initial and follow-up visits [14] in both the community and hospital settings to ensure that all prescribed medicine has an appropriate indication.

Pharmacists could optimise medication therapy by reviewing prescriptions in the pharmacy or ward setting; however, the effect could be greater when pharmacists are made part of ward round discussions [3] where medication errors could be identified sooner. Through the medication reconciliation process and prescription review, pharmacists could identify dose omissions and MRPs [3]. An MRP is an undesirable event that includes:

1. An undesirable event or risk of an event,
2. Medication therapy of the patient, and
3. A relationship between the undesirable event and medication therapy [39].

Table 5 summarises the problems related to medicine.

Further, medication use could be improved through optimising medication labels [40] for inpatient use (e.g., reconstitution of intravenous therapy and stability once reconstituted) or outpatient use (e.g., being specific in duration of therapy for antibiotics, “store in fridge” if it is required, give 5 mL (1 medicine spoon)). Counselling and education are vital roles fulfilled by pharmacists [11] that might optimise medication therapy.

### Education and Counselling

Pharmacists could educate carers and healthcare workers. Counselling sessions should include indication of the prescribed medicine, administration directions, storage instructions, side effects, and adverse effects [1,11]. Directions for medicine use should be clear and specific. Directions should include the correct dose (e.g., 5 mL = 1 medicine spoon), method and route of administration (crush, chew), frequency of administration (i.e., dosage) (e.g., every 12 h or 8 h), when to administer the medicine (e.g., after a meal for pain and fever), and the duration (e.g., for 5 d) [1,11]. Additional counselling points to include (if applicable) are avoiding sunlight, refrigerated storage, and/or shaking the bottle before use [1,38].

Pharmacists could implement the following in their counselling sessions to empower carers to optimally use medication:

- explaining the instructions to administer the medicines,
- providing a practical demonstration of how to administer the medicines, and
- marking the appropriate dose on the measuring equipment.

Another method is to colour code the medicine, especially when various medication solutions are administered (e.g., with paediatric ART) [11].

Pharmacists should educate and train various healthcare professionals on paediatric dosing. In hospital settings, how to mix intravenous preparations could be taught by pharmacists [40]. When dispensing medicine, the pharmacist should always ask the carer or health care worker if they have any questions. Additionally, pharmacists should educate carers on safe antibiotic use to combat antimicrobial resistance [41].

It is crucial that the main carer (parent/guardian) is involved in the patient’s care plan. Careful medication reconciliation on admission is critical, and any dose changes, addition, or discontinuation of medicines should be shared with the carer. This is especially important if the carer is administering medicines while the paediatric patient is in hospital [21].

To prevent the spread of infection, washing hands should be imperative when working with medication. The washing of hands should be performed according to the National Hand Hygiene Initiative and the World Health Organization’s “My 5 Moments for Hand Hygiene” [42]. The “My 5 Moments for Hand Hygiene” approach recommends washing hands with either soap, water, or alcohol-based hand rub.

## 6. Conclusions

Pharmacists have a significant role to play in optimising medication therapy and paediatric patient safety in both the community and hospital settings. Following the basic principles could lead to safe medication use in paediatric patients and achieve health outcomes.

## Figures and Tables

**Table 1:** Definition of paediatric patient terms [23,24,25].

Term	Definition
Adolescent	Patient between the ages of 12 to 21 years
Child	Paediatric patient between the ages of 2 to 12 years
Infant	Paediatric patient between the ages of 0 to 24 months
Neonate	Paediatric patient from birth to 28 days of age
Preterm	Neonate born before 37 weeks of gestation
Term	Neonate born at 39 weeks of gestation
Toddler	Paediatric patient between the ages of 1 and 2 years

**Table 2:** Common medicines prescribed in infants [17,27,29,33,34].

Medicine	Product Concentration	Calculating the Dose
amoxicillin (PO)	125 mg/5 mL	125 mg/5 mL
	250 mg/5 mL	250 mg/5 mL
ferrous gluconate (PO)	350 mg/5 mL (elemental iron 8 mg/1 mL)	8 mg/1 mL 40 mg/5 mL
ferrous lactate (PO)	15 mg/0.6 mL	15 mg/0.6 mL
ibuprofen (PO)	100 mg/5 mL	100 mg/5 mL
paracetamol (PO)	120 mg/5 mL	120 mg/5 mL
vitamin D (PO)	200 units/1 drop	200 units/1 drop
amoxicillin/clavulanic acid (PO)	125 mg/31.25 mg/5 mL	125 mg/5 mL
	250 mg/62.5 mg/5 mL	250 mg/5 mL
amoxicillin/clavulanic acid (IV)	500 mg/100 mg	500 mg
	1000 mg/200 mg	1000 mg
piperacillin/tazobactam (IV)	4000 mg/500 mg	4000 mg
trimethoprim/sulfamethoxazole (PO)	40 mg/200 mg/5 mL	40 mg/5 mL
	80 mg/400 mg/tablet	80 mg/1 tablet
trimethoprim/sulfamethoxazole (IV)	80 mg/400 mg/5 mL	80 mg/5 mL

**Table 3:** Medicines cleared by kidneys [18,35].

Class	Medicine Name
Antibiotics	amikacin, amoxicillin, cefazolin, cefepime, cefotaxime, cefuroxime, ceftazidime, gentamicin, meropenem, piperacillin/tazobactam, sulfamethoxazole, tobramycin, vancomycin
Anti-fungal	amphotericin B, fluconazole
Anti-hypertensives	enalapril, milrinone, verapamil
ART	emtricitabine, tenofovir disoproxil fumarate, lamivudine, zidovudine
Anti-TB	ethambutol

**Table 4:** Hepatically cleared medicine [18,35].

Class	Medicine Name
Anti-epileptics	carbamazepine, phenytoin, sodium valproate
Anti-TB	isoniazid, pyrazinamide, rifampicin
ART	efavirenz, nevirapine
Other	cyclosporine, paracetamol

**Table 5:** Examples of medication-related problems (MRP) [39].

MRP	Example	Identifying MRP
Unnecessary medicine	Patient is receiving unnecessary therapy with paracetamol. Explanation: Patient experiences no pain; thus, not indicated.	Review indication Ensure non-duplication
Additional therapy required	Patient requires additional therapy for the prevention of TB. Explanation: Paediatric patient with family member with TB.	Review indication/current diagnosis
Dosage too high	Ibuprofen dosage prescribed is too high to manage pain. Explanation: Ibuprofen is prescribed at a higher dosage than recommended (5 mg/kg/dose to 10 mg/kg/dose, every 6 h to 8 h).	Review dosage prescribed
Alternative therapy	Patient requires alternative therapy for treating infection. Explanation: Patient requires alternative antibiotic.	Review indication/prescribedmedicine.
Adverse drug reaction	Patient requires slower dose escalation of lamotrigine. Explanation: Patient experienced severe skin rash with increase in lamotrigine dose.	Evaluate patient outcome. Determine if adverse effect is due to patient’s medicine.
Non-adherence	Patient requires a liquid formulation of sodium valproate to prevent seizures. Explanation: Patient is unable to swallow.	Evaluate patient outcome. Determine if outcomes are achieved or not. Determine challenges.
